# The Optimization of the Dehydration Temperature of Peels from Prickly Pears

**DOI:** 10.3390/foods14050811

**Published:** 2025-02-26

**Authors:** Olimpia Panza, Matteo Alessandro Del Nobile, Amalia Conte

**Affiliations:** 1 Department of Humanistic Studies, Letters, Cultural Heritage and Educational Sciences, University of Foggia, Via Arpi, 176, 71121 Foggia, Italy; olimpia.panza@unifg.it (O.P.); amalia.conte@unifg.it (A.C.); 2 Department of Economics, Management and Territory, University of Foggia, Via A. da Zara, 11, 71122 Foggia, Italy

**Keywords:** prickly pear by-product, dehydration, energy consumption, evaporation, kinetic

## Abstract

The optimization of the prickly pear peel (PPP) dehydration temperature was addressed. Two indicators of efficiency were used to select the optimal dehydration temperature: one related to the process productivity, another to the energy consumption. To calculate them the PPP dehydration kinetics were measured at three different temperatures (i.e., 50, 60, and 70 °C) along with the energy consumption of the process. A mathematical model was used to fit the dehydration kinetics. The influence of the temperature on the kinetics was assessed by analyzing the dependence of the fitting parameters on the dehydration temperature. It was found that both the kinetic parameters and the equilibrium parameter depend on the temperature through an exponential-type equation. The model was also used to calculate both the process productivity and the average energy consumed by the dehydration cabinet per grams of evaporated water when 99% dehydration is reached. The two efficiency indicators suggested that the optimal drying temperature is 70 °C, both being the indicators decreasing function of the temperature.

## 1. Introduction

In recent years, food waste has increasingly become a global problem. The agri-food sector is one of the most responsible since by-products from fruit and vegetable processing are very abundant despite containing numerous bioactive molecules [1]. Recently, recycling and sustainable exploitation of by-products has drawn attention to the circular bioeconomy for the production of high-value products [2,3,4]. In this context, prickly pears can play an important role [5]. Prickly pears (*Opuntia ficus-indica* L. Mill.) are consumed as fresh fruit or for preparation of jams, alcoholic beverages, soft drinks, syrups, candied fruit, and flour. The peel of the fruit, which represents about 48% of the entire fruit, is generally discarded, and considering its large amount, the management of fruit by-products became a great problem from both the economic and environmental point of view [6,7]. Prickly pear peels are a promising source of phenolic compounds, proteins, minerals, dietary fibers, and vitamins [8,9,10]. For these recognized properties, by-products from prickly pears and their extracts were used as ingredients to enrich food [11,12,13,14] or promote their better preservation [15].

Due to the high level of moisture and fermentable carbohydrates, peels from prickly pears cannot be stored for long periods [16]. The most common method for their preservation is the use of a dehydration process, with the main objective to remove water up to a certain level at increasingly faster rates, optimizing energy expenditure [17]. A small number of authors have previously studied the impact of different drying methods on fruits and peels from *O. ficus-indica*. Ovens, freeze-drying, and microwaves were found as main drying techniques adopted on prickly pear by-products, and the nutritional composition of dried peels and their utilization as flour supplements were investigated more than the impact of drying methods [18,19]. There is a study that examines the effect of drying air conditions on the drying kinetics of prickly pear fruit, and not the by-products, in a convective solar drier operating with an auxiliary heating system under air-controlled conditions [20]. More recently, Abraham-Juárez et al. [21] evaluated the effect of air temperature and sample thickness on drying kinetics of prickly pear fruit paste, prepared by blending concentrated juice, prickly pear peel flour (4%), and citric acid (0.15%), disposed in a thin layer. Therefore, there is a notable gap in the existing literature regarding the drying of *O. ficus-indica* peel from a kinetics point of view that justifies the novelty of the current research. Generally, dehydration is effective to prevent microbiological proliferation, even though the drying method can affect the composition, physicochemical properties, and prebiotic potential of dietary fiber concentrates from fruit peels [22]. Studying the dehydration kinetics of by-products was useful to determine the best time to stop the dehydration. Mathematical models of dehydration are important in the design and optimization of processing operations because they can describe the mechanisms and the influence that certain process variables exert upon moisture transfer [23,24]. Some efforts in the scientific literature to develop mathematical models able to describe the dehydration kinetics have been made using other food by-products, such as grape pomace [25] and tomato seeds and peels [26]. These authors first compared more than one mathematical model and demonstrated that the Dual-Stage Type A model is the best one to describe the dehydration kinetic of grape pomace, considering both the suitability and complexity of the mathematical approach adopted [25]. The same model was also applied with success to describe the dehydration kinetic of tomato peels and seeds, thus demonstrating the influence of dehydration on temperature and also calculating the dehydration rate as a function of tomato by-product water content [26]. Much later, Lordi et al. [27] assessed the productivity of the dehydration considering the time needed to complete the drying process. The authors demonstrated that the productivity of the dehydration of tomato peels and seeds increased as the dehydration temperature increased. The scarcity of publications on prickly pear peel dehydration, coupled with the absence of previous investigations into the influence of drying temperature on the energy consumption, underscore the imperative for further research that is aimed to study the dehydration kinetics of these by-products, being prodromic to an optimization of the dehydration process. Therefore, the current study is aimed to find the most convenient temperature in terms of dehydration efficiency. Based on the background already available, in the current study, the mathematical model known as the Dual-Stage Type A model [25] was used to describe the dehydration kinetic of prickly pear peels (PPPs) to the final aim of finding the best temperature among 50, 60, and 70 °C. Both the productivity of the process, i.e., the time required to reach 99% complete dehydration [27], and the energy consumption by the dehydration cabinet per grams of evaporated water were also assessed.

## 2. Materials and Methods

### 2.1. Prickly Pear Peel Dehydration Process

Red prickly pears (*O. ficus-indica* L. Mill.), cultivar Sanguigna, were kindly provided by a local dealer (Manfredonia, Puglia, Italy), transported to the laboratory, and stored at 4° C prior to processing. Then, the fruits were peeled as reported in the study of Panza et al. [15] and the prickly pear peels (PPPs) were dried. A conventional dryer (PF–SIC CO80PRO, SICCOTECH, Campobasso, Italy) was used to carry out a hot air-drying process using natural convection at room pressure. The dryer was a cabinet with a volume of 0.6 m^3^, able to contain 20 racks (72 × 53 × 3 cm). PPPs were dehydrated at 50 °C, 60 °C, and 70 °C, setting the relative humidity at 5%. About 1 kg of fresh by-products was distributed on different racks of the cabinet to form a uniform and thin layer. The three temperatures were selected based on a previous research study carried out on tomato peels and seeds, where it was demonstrated that dehydration temperatures ranging between 50 and 70 °C did not affect the antimicrobial and nutritional quality of the dehydrated powder [27]. When the weight of dehydrated PPPs was constant for several consecutive measurements, the drying process was stopped. During dehydration, the weight was recorded periodically on a PPP sample of 5 g by a thermal balance (Sartorius, Göttingen, Germany) set at 130 °C. Every time, the sample was removed from the drying cabinet in a few seconds, thus making it reasonable to assume that the sampling procedure did not affect the dehydration kinetics. A total of 11 samplings was carried out.

### 2.2. Prickly Pear Peel Water Concentration

The following, Equation (1), was used to calculate the PPP water concentration:

(1) $$C\left(t\right)=\frac{W\left(t\right)-W_{F}}{W_{F}}\cdot 100$$

where $C\left(t\right)$ is the sample water concentration at time t expressed as $\left[\frac{g\;water}{100\;g\;dry\;matter}\right]$, $W\left(t\right)$ is the weight of sample at time t expressed as $\left[g\right]$, $W_{F}$ is the weight expressed as $\left[g\right]$ of the sample after it has been kept at 130 °C and all the water was desorbed.

The amount of water desorbed at time t $\left(M_{H_{2}O}\left(t\right)\right)$ was calculated according to the following equation:

(2) $$M_{H_{2}O}\left(t\right)=C_{0}-C\left(t\right)$$

where $C_{0}$ is the initial sample water concentration. The amount of water desorbed at equilibrium $\left(M_{H_{2}O}^{\infty }\right)$ was estimated according to the following expression:

(3) $$M_{H_{2}O}^{\infty }=C_{0}-C_{\infty }$$

where $C_{\infty }$ is the sample water concentration at the end of the dehydration process.

### 2.3. Dehydration Kinetic

The model proposed in the current study to describe PPP dehydration kinetics was that proposed by Conte et al. [25]. Based on what is reported in the above paper, the following relationships can be used to describe the PPP dehydration kinetics:

(4) $$0\;\leq \;t\;\leq \;t_{c}\;M_{H_{2}O}\left(t\right)=K_{1}\cdot t$$

(5) $$t>t_{c}\;M_{H_{2}O}\left(t\right)=K_{1}\cdot t_{c}+K_{2}\cdot \left\{1-exp\left[-\left(t-t_{c}\right)\cdot \left(\frac{K_{1}}{K_{2}}\right)\right]\right\}$$

where $K_{1}$ is the desorption rate during the 1st stage $\left[\frac{g\;desorbed\;water}{100\;g\;dry\;matter}\cdot \frac{1}{min}\right]$, $K_{2}$ is maximum amount of water desorbed during the 2nd stage $\left[\frac{g\;desorbed\;water}{100\;g\;dry\;matter}\right]$, t is the time $\left[min\right]$, and *t_c_* is the moment in which the transition from one stage to another takes place. It is worth noting that the total amount of water desorbed at equilibrium $\left(M_{H_{2}O}^{\infty }\right)$ is equal to $K_{1}\cdot t_{c}+K_{2}$, whereas the ratio $\frac{K_{1}}{K_{2}}$ is the dehydration kinetic constant related to the 2nd stage.

The goodness of fit was expressed by the mean relative deviation modulus $\left(\bar{E}\%\right)$ [28]:

(6) $$\bar{E}\%=\frac{100}{N}\cdot \sum_{i=1}^{i=N}\frac{\left|M_{i}^{exp}-M_{i}^{pred}\right|}{\left|M_{i}^{exp}\right|}$$

where N is the number of experimental data, $M_{i}^{exp}$ is the experimental value, and $M_{i}^{pred}$ is the predicted value.

The dehydration rate $\left(\frac{{dM}_{H_{2}O}\left(t\right)}{dt}\right)$ can be easily obtained by taking the time derivative of Equations (4) and (5):

(7) $$0\;\leq \;t\;\leq \;t_{c}\;\frac{{dM}_{H_{2}O}\left(t\right)}{dt}=K_{1}$$

(8) $$t>t_{c}\;\frac{{dM}_{H_{2}O}\left(t\right)}{dt}=K_{1}\cdot exp\left[-\left(t-t_{c}\right)\cdot \left(\frac{K_{1}}{K_{2}}\right)\right]$$

Combining Equations (2), (4), (5), (7), and (8), it is possible to correlate $\frac{{dM}_{H_{2}O}\left(t\right)}{dt}$ to $C\left(t\right)$:

(9) $$\left({C_{0}-K}_{1}\cdot t_{c}\right)\;\leq \;C\left(t\right)\;\frac{{dM}_{H_{2}O}\left(t\right)}{dt}={\;K}_{1}$$

(10) $$\left[C_{0}-\left(K_{1}\cdot t_{c}+K_{2}\right)\right]\leq C\left(t\right)<\left({C_{0}-K}_{1}\cdot t_{c}\right)\;\frac{{dM}_{H_{2}O}\left(t\right)}{dt}=\frac{K_{1}}{K_{2}}\cdot \left[K_{1}\cdot t_{c}+K_{2}-C_{0}\right]+\frac{K_{1}}{K_{2}}\cdot C\left(t\right)$$

To describe the dependence of the parameters of Equations (4) and (5) on the testing temperature, an Arrhenius-type equation has been used, as reported in the following:

(11) $$ln\left(Y\left(T\right)\right)=ln\left(Y_{0}\right)-\frac{Y_{1}}{R}\cdot \frac{1}{T}$$

where $Y\left(T\right)$ is the parameter value at the temperature T, $Y_{0}$ is the pre-exponential term, $Y_{1}$ is a model’s parameter whose physical meaning depends on the physical quantity $Y\left(T\right)$, and R is the universal gas constant.

### 2.4. Dehydration Process Productivity

As reported in a previous paper [27], the productivity of the dehydration process can be linked to the time necessary to reach a given extent of dehydration (ext%), named $t_{ext\%}$, whereas the extent of dehydration is defined according to Equation (12):

(12) $$ext\%\left(t\right)=\frac{M_{H_{2}O}\left(t\right)}{M_{H_{2}O}^{\infty }}\cdot 100$$

Combining Equations (4), (5), and (12), it was possible to relate $t_{ext\%}$ to ext%:

(13) $$0\;\leq \;ext\%\;\leq \;\frac{K_{1}\cdot t_{c}}{M_{H_{2}O}^{\infty }}\cdot 100\;t_{ext\%}=\frac{M_{H_{2}O}^{\infty }}{K_{1}\cdot 100}\cdot ext\%$$

(14) $$ext\%>\frac{K_{1}\cdot t_{c}}{M_{H_{2}O}^{\infty }}\cdot 100\;t_{ext\%}=t_{c}-ln\left(\frac{K_{1}\cdot t_{c}+K_{2}-ext\%\cdot \frac{M_{H_{2}O}^{\infty }}{100}}{K_{2}}\right)\cdot \frac{K_{2}}{K_{1}}$$

### 2.5. Energy Consumption of the Dehydration Process

To determine the optimal dehydration temperature in terms of energy consumption, the average energy consumed by the dehydration cabinet at time t per grams of evaporated water $\left(\bar{E}\left(t\right)\right)$, the following expression was used:

(15) $$\bar{E}\left(t\right)=\frac{\hat{E}\left(t\right)}{M_{H_{2}O}\left(t\right)}$$

where $\bar{E}\left(t\right)$ is expressed as $\left[\frac{kW\cdot h}{g\;evaporated\;water}\right]$ and $\hat{E}\left(t\right)$ is the energy consumed by the dehydration cabinet at time t per 100 g of dry matter, expressed as $\left[\frac{kW\cdot h}{100\;g\;dry\;matter}\right]$. The value of $\hat{E}\left(t\right)$ is defined as reported in the Expression (16):

(16) $$\hat{E}\left(t\right)=\frac{E\left(t\right)}{m_{Tot}\cdot x_{dm}}$$

where $E\left(t\right)$ is the energy consumed by the dehydration cabinet at time t, expressed in $\left[kW\cdot h\right]$; $m_{Tot}$ is the initial total mass in the dehydrator; $x_{dm}$ is the initial PPP weight fraction of dry matter; and $x_{dm}$ was calculated as $\frac{m_{dm}}{m_{Tot}}$, where $m_{dm}$ is the total mass of dry matter in the dehydrator. $E\left(t\right)$ was calculated according to Equation (17):

(17) $$E\left(t\right)=\Theta \cdot t$$

where Θ is the measured power supplied to the dehydration cabinet per 100 g dry matter, expressed as $\left[\frac{kW\cdot h}{min\cdot 100\;g\;dry\;matter}\right]$. Combining Equations (4), (5), (12), (15)–(17), it was possible to relate $\bar{E}$ to ext% through the following relations:

(18) $$0\;\leq \;ext\%\;\leq \;\frac{K_{1}\cdot t_{c}}{M_{H_{2}O}^{\infty }}\cdot 100\;\bar{E}\left(ext\%\right)=\frac{\Theta }{K_{1}}$$

(19) $$\frac{K_{1}\cdot t_{c}}{M_{H_{2}O}^{\infty }}\cdot 100<ext\%\leq 100\;\bar{E}\left(ext\%\right)=\frac{\Theta \cdot \left\{t_{c}-\frac{ln\left[\frac{\left[1-\frac{ext\%}{100}\right]\cdot M_{H_{2}O}^{\infty }}{K_{2}}\right]}{\left(\frac{K_{1}}{K_{2}}\right)}\right\}}{ext\%}\cdot \frac{100}{M_{H_{2}O}^{\infty }}$$

## 3. Results and Discussion

The optimization of the dehydration temperature of PPPs was addressed by studying the dehydration kinetics of PPPs at three different temperatures, along with the energy consumption of the process. The optimal dehydration temperature was evaluated by considering both the productivity of the process and its environmental impact in terms of energy consumption.

### 3.1. Dehydration Kinetics

**Figure 1:**
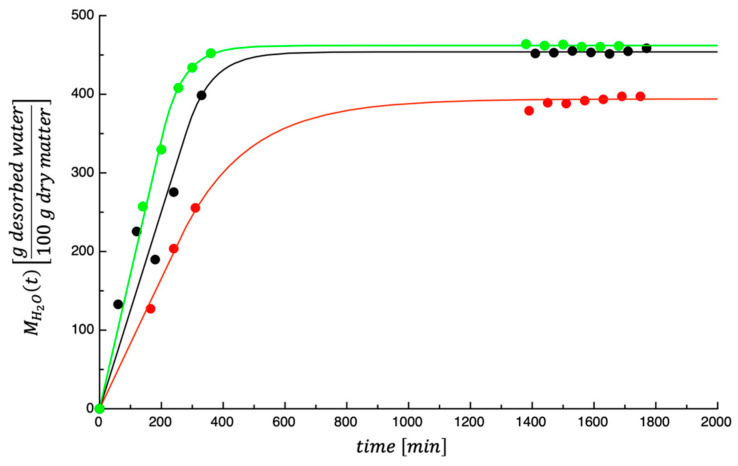
Dehydration kinetics of prickly pear by-products at 50 °C (red), 60 °C (black), and 70 °C (green). The curves shown in the figure were obtained by fitting Equations (4) and (5) to the experimental data.

Figure 1 shows the dehydration kinetics of the investigated by-products at three different temperatures. As would be expected, in the initial phase of dehydration, the amount of desorbed water increased steadily over time, and then, it gradually approached to its equilibrium value. By fitting the Equations (4) and (5) to the experimental data, the curves shown in Figure 1 and the fitting parameters reported in Table 1 were obtained. As expected, the three temperatures greatly influenced these values. Concerning the goodness of fit, $\bar{E}\%$ values of 1.49, 8.28, and 1.16 were obtained for the tests performed at 50, 60, and 70 °C, respectively. The calculated $\bar{E}\%$ at 50 and 70 °C can be considered very low, while the one obtained at 60 °C is slightly high. However, as can be seen from data shown in Figure 1, the set collected at 60 °C is slightly more scattered than the other two sets of data. This is most probably the reason why the $\bar{E}\%$ obtained at 60 °C is higher than that obtained at 50 and 70 °C. Based on this experimental evidence, it can be stated that the proposed model can be used to describe the dehydration kinetics of PPPs in the range of the investigated temperatures.

**Figure 2:**
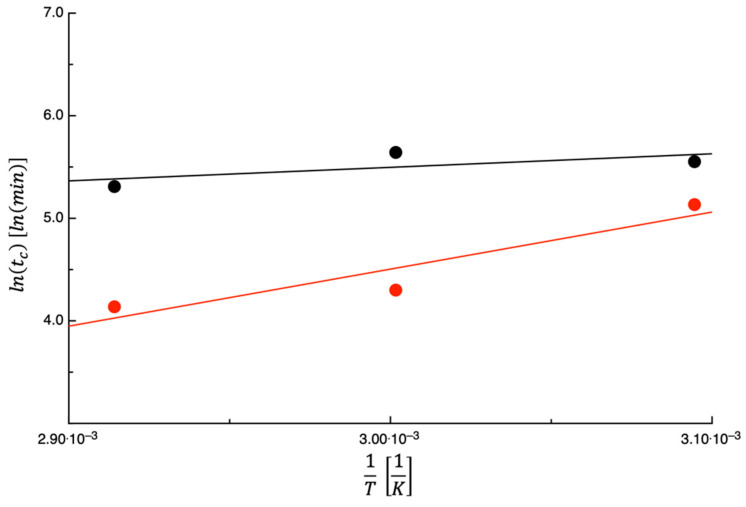
The expression $ln\left(t_{c}\right)$ plotted as a function of $\frac{1}{T}$ for prickly pear peels (black) and tomato peels (red). The straight lines are obtained by fitting Equation (7) to the data.

The dependence of the parameters $t_{c}$, $K_{1}$, $\frac{K_{1}}{K_{2}}$, and $M_{H_{2}O}^{\infty }$ $\left(i.e.,\;K_{1}\cdot t_{c}+K_{2}\right)$ on the temperature was also assessed. To the aim, Figure 2 shows the $ln\left(t_{c}\right)$ plotted as a function of $\frac{1}{T}$. As can be inferred from the figure, as the temperature increases, $t_{c}$ decreases. According to Conte et al. [25], the observed trend is not unexpected. In fact, these authors assumed that during the 1st stage, the water evaporation rate from the by-product surface is the main factor limiting the dehydration rate. The water evaporation depends on both the enthalpy of water vaporization and on the convective exchange coefficient. When the dehydration temperature increases, the two phenomena have different behavior, and the vaporization enthalpy decreases, whereas the convective heat exchange coefficient presumably does not change to a great extent. Consequently, in the 1st stage, the dehydration rate should increase with the temperature, while the parameter $t_{c}$, as the time in which the transition from one dehydration mechanism to another occurs, is supposed to decrease.

The straight line in Figure 2 was obtained using Equation (7); the values of $ln\left(Y_{0}\right)$ and $\frac{Y_{1}}{R}$ obtained are listed in Table 2 along with the value of $\bar{E}\%$. As the $\bar{E}\%$ values listed in the table suggest, Equation (7) describes quite adequately the dependence of $t_{c}$ on the temperature. Unfortunately, no data on the dehydration kinetics of PPPs and its dependence on the drying temperature are available in the literature. Comparison with other works focused on the dehydration of by-products is not easy. In fact, the physical quantity used in the current study for the dehydration kinetics is different from those used in other papers. In this manuscript the amount of desorbed water per gram of dry matter is used, whereas other researchers used either the water content on dry base or the moisture ratio [24]. Even more important, the models used by other researchers to describe the dehydration kinetics are different from that used in this study. The comparison between two sets of data is performed by comparing the parameters of the model used to fit the data. Considering that the model used in the current manuscript was introduced very recently, there is only a manuscript that used the same approach. Therefore, the sole possible comparison is with the work of Lordi et al. [27], dealing with the dehydration of tomato peels and seeds (TPSs). For comparative purpose, in Figure 2, the TPS data are also shown. As can be inferred from what is reported in the figure, TPSs show a trend like that of PPPs, as both sets of data decrease with temperature; however, $t_{c}$ values measured for TPSs are always lower than that of PPPs. Considering that PPPs and TPSs have an initial water content of $439.1\pm 30.2\frac{g\;water}{100\;g\;dry\;matter}$ and $217.8\pm 5.47\frac{g\;water}{100\;g\;dry\;matter}$, respectively, the lower $t_{c}$ values observed for TPSs may be because TPSs have a lower initial water content.

**Figure 3:**
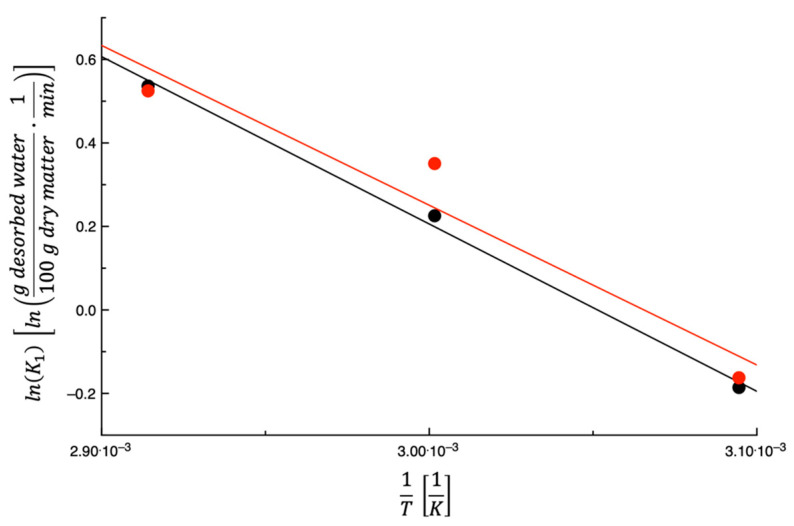
The expression $ln\left(K_{1}\right)$ plotted as a function of $\frac{1}{T}$ for prickly pear peels (black) and tomato peels (red). The straight lines are obtained by fitting Equation (7) to the data.

Figure 3 shows $ln\left(K_{1}\right)$ plotted as a function of the inverse of the dehydration temperature; as can be seen in the figure, $K_{1}$ increases as the temperature increases. This is not surprising considering that $K_{1}$ is the desorption rate during the 1st stage. In fact, as reported beforehand, the enthalpy of the vaporization of water and the convective exchange coefficient are two factors that influence the water evaporation rate from the food surface. When increasing the dehydration temperature, the vaporization enthalpy decreases, whereas the convective exchange coefficient presumably remains almost the same. Therefore, it is expected that $K_{1}$ increases with the temperature. The straight lines shown in the figure are the best fit of Equation (7) to the data. The fitting parameters are listed in Table 2, along with the $\bar{E}\%$ values. As can be inferred from data shown in the figure and the $\bar{E}\%$ values calculated, an Arrhenius-type equation can indeed be used to describe the dependence of $K_{1}$ on the temperature.

For the sake of comparison, the data of Lordi et al. [27] on TPSs are also shown in Figure 3. As can be inferred from what is reported in the figure, there is a small difference between the two sets of data, most probably due to the fact that this work and that of Lordi et al. [27] used the same dehydrator and the same setup. This means that the rate at which the water evaporates from the surface is very similar for these two by-products.

**Figure 4:**
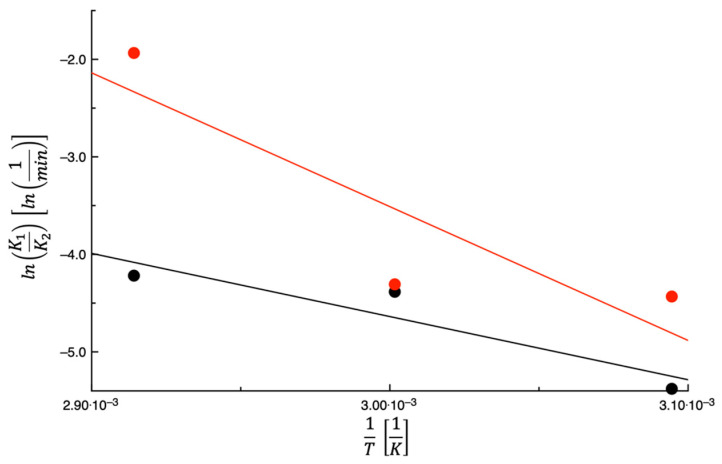
The expression $ln\left(\frac{K_{1}}{K_{2}}\right)$ plotted as a function of $\frac{1}{T}$ for prickly pear peels (black) and tomato peels (red). The straight lines are obtained by fitting Equation (7) to the data.

Figure 4 shows $ln\left(\frac{K_{1}}{K_{2}}\right)$ plotted as a function of $\frac{1}{T}$. As found in the case of $K_{1}$, a steady increase with the temperature was also observed in the case of $\frac{K_{1}}{K_{2}}$. This trend is not surprising, as $\frac{K_{1}}{K_{2}}$ is the 2nd-stage kinetic constant. As reported by Lordi et al. [27], $\frac{K_{1}}{K_{2}}$ is strictly related to the diffusion of water toward the food surface. Considering that the diffusion of low molecular weight compounds is an activated process, it is expected that $\frac{K_{1}}{K_{2}}$ is an increasing function of the temperature. The straight line of Figure 4 was obtained by fitting Equation (7) to the data. In Table 2, the fitting parameter and the $\bar{E}\%$ value are listed. It is worth noting that the value of $\bar{E}\%$ is low, thus signifying that an Arrhenius-type equation can be used to describe the dependence of $\frac{K_{1}}{K_{2}}$ on the temperature.

The data of Lordi et al. [27] on TPSs are also shown in Figure 4 for comparative purposes. According to the data shown in the figure, the $\frac{K_{1}}{K_{2}}$ of PPPs is always lower than that of TPSs, meaning that the water diffusion toward the by-product surface takes longer in the case of PPPs. There are two factors that both contribute to determining the value of $\frac{K_{1}}{K_{2}}$: the diffusion coefficient of the water through the by-product, which is an increasing function of the water content, and the distance that the water molecules cover to reach the surface. As reported beforehand, the initial water content of PPPs is almost double that of TPSs; however, the tomato skin is much thinner than the prickly pear peel. Data suggest that the distance that the water molecules cover to reach the surface prevails on the water diffusion coefficient.

**Figure 5:**
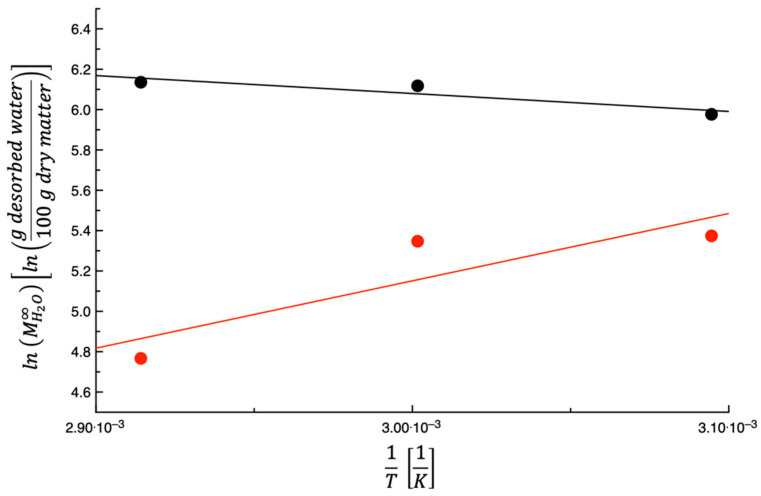
The expression $ln\left(M_{H_{2}O}^{\infty }\right)$ plotted as a function of $\frac{1}{T}$ for prickly pear peels (black) and tomato peels (red). The straight lines are obtained by fitting Equation (7) to the data.

Figure 5 shows $ln\left(M_{H_{2}O}^{\infty }\right)$ plotted as a function of the inverse of the testing temperature. As can be seen in the figure, when the temperature increases, the quantity of desorbed water at equilibrium also increases. Considering that the PPP water concentration at the end of the dehydration process is related to $M_{H_{2}O}^{\infty }$ through Equation (3), it can be stated that by increasing the temperature, the water concentration at the end of the dehydration process decreases. In other words, an exothermic process occurs with water solubilization in PPPs. The straight line shown in Figure 5 was obtained by fitting Equation (7) to the data. Table 2 lists the values of both Equation (7) parameters and $\bar{E}\%$. A small $\bar{E}\%$ was found, thus indicating that Equation (7) can be used to describe the data.

As conducted beforehand, the data of Lordi et al. [27] on TPSs were used for comparative purposes; they are also shown in Figure 5. As can be inferred from data shown in the figure, for TPSs, $M_{H_{2}O}^{\infty }$ is a decreasing function of the temperature. In other words, the TPS equilibrium water concentration increases with the temperature, indicating that the water solubilization in TPSs is an endothermic process. Figure 5 shows that PPPs and TPSs have different behavior when considering the water solubilization process; the former is exothermic, while the latter is endothermic. A possible explanation for what was observed is that the PPPs and the TPSs have different chemical compositions [29,30]. In PPPs, the hydrophilic moiety prevails over the hydrophobic one, while in the case of TPSs, the opposite occurs. In fact, the initial water content of PPPs is almost double than that of TPSs.

**Figure 6:**
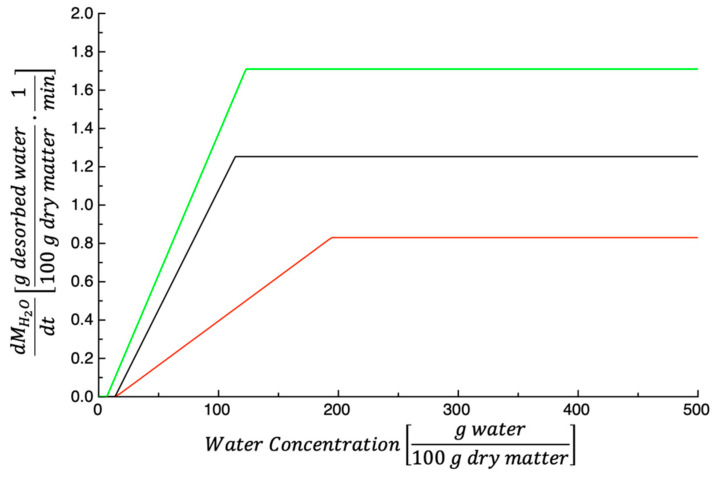
The dependence of the dehydration rate $\left(\frac{{dM}_{H_{2}O}\left(t\right)}{dt}\right)$ on water concentration at 50 °C (red), 60 °C (black), and 70 °C (green).

The dependence of the dehydration rate $\left(\frac{{dM}_{H_{2}O}\left(t\right)}{dt}\right)$ on $C\left(t\right)$ was calculated according to Equations (9) and (10). The values of the model’s parameters used are those listed in Table 1, the results are shown in Figure 6. As expected, $\frac{{dM}_{H_{2}O}\left(t\right)}{dt}$ is strongly affected by the drying temperature. In fact, whatever the PPP water concentration, the dehydration rate increases with the temperature.

**Figure 7:**
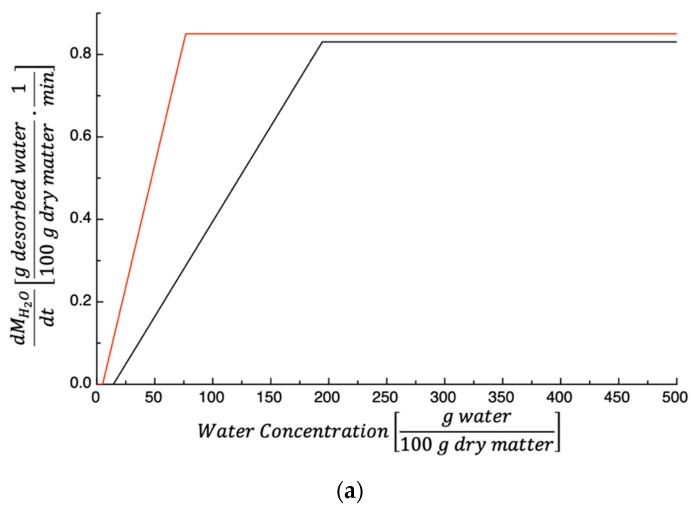

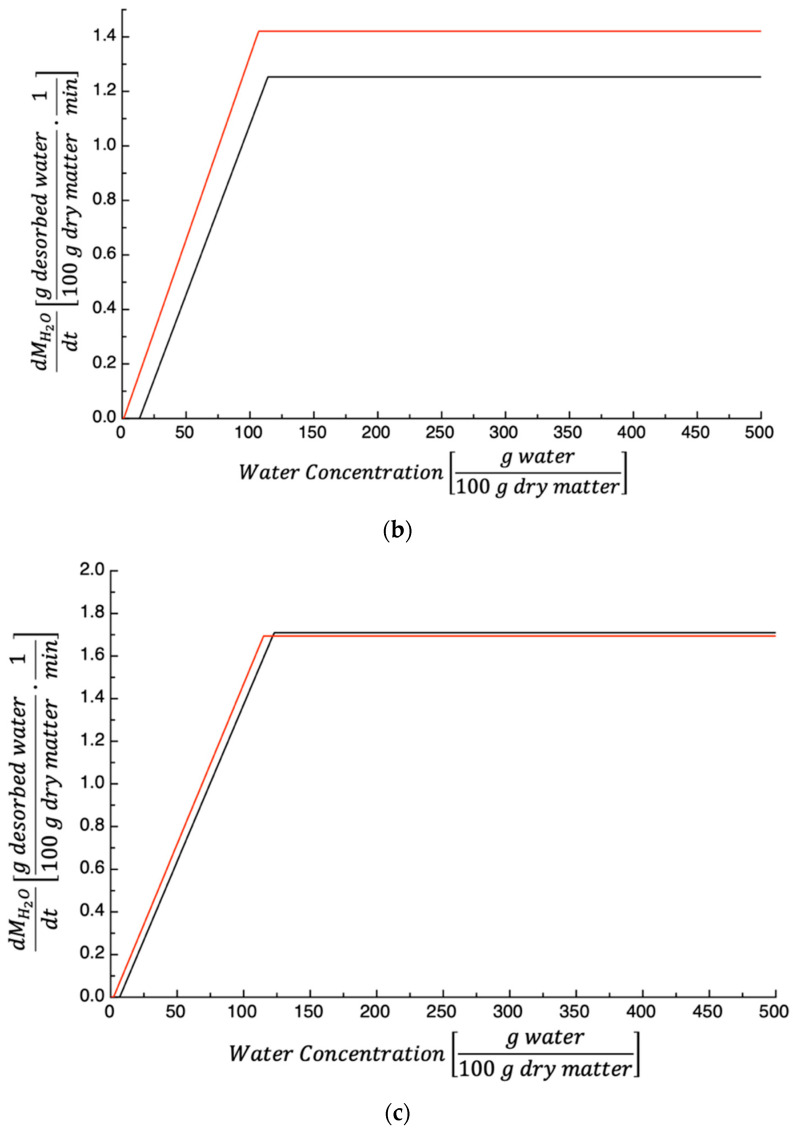
(**a**) The dependence of the dehydration rate $\left(\frac{{dM}_{H_{2}O}\left(t\right)}{dt}\right)$ at 50 °C on the water concentration of prickly pear peels (black) and tomato peels (red). (**b**) The dependence of the dehydration rate $\left(\frac{{dM}_{H_{2}O}\left(t\right)}{dt}\right)$ at 60 °C on the water concentration of prickly pear peels (black) and tomato peels (red). (**c**) The dependence of the dehydration rate $\left(\frac{{dM}_{H_{2}O}\left(t\right)}{dt}\right)$ at 70 °C on the water concentration of prickly pear peels (black) and tomato peels (red).

For the sake of comparison, the dehydration rate of the investigated by-product was compared to that of TPSs [27]. The results are shown in Figure 7. Figure 7a shows $\frac{{dM}_{H_{2}O}\left(t\right)}{dt}$ plotted as a function of water concentration; the data shown refer to a dehydration temperature of 50 °C. As can be inferred from the figure, the TPS dehydration rate is always higher than that of PPPs. This is not surprising considering that $K_{1}$ and $\frac{K_{1}}{K_{2}}$ values of TPSs are larger than that of PPPs (see data shown in Figure 3 and Figure 4).

Figure 7b shows the dehydration rate vs. $C\left(t\right)$; in this case, the drying temperature is 60 °C. As discussed previously, the data shown in the figure are not surprising considering that the values of $K_{1}$ and $\frac{K_{1}}{K_{2}}$ found for TPSs are larger than that of PPPs (see data shown in Figure 3 and Figure 4).

Figure 7c shows $\frac{{dM}_{H_{2}O}\left(t\right)}{dt}$ vs. $C\left(t\right)$, and the drying temperature is 70 °C. In this case, the initial PPP dehydration rate is lightly higher than that of TPSs, in accordance with what is reported in Figure 3. However, during the 2nd stage, the contrary is true (see data shown in Figure 4).

### 3.2. Dehydration Process Productivity

**Figure 8:**
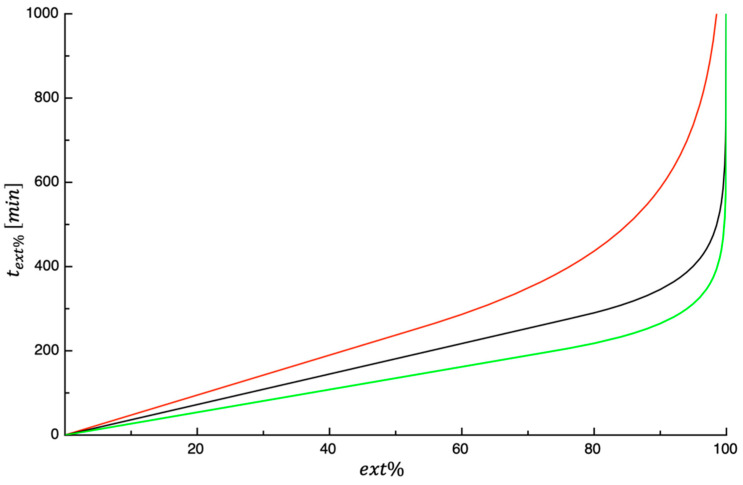
The variable $t_{ext\%}$ plotted as a function of ext%. The curves referring to the three investigated temperatures (50 °C red, 60 °C black, and 70 °C green) are predicted using Equations (13) and (14).

Figure 8 shows $t_{ext\%}$ plotted as a function of ext%. The curves displayed in the above-mentioned figure were predicted using Equations (13) and (14), and the values of parameters used to predict the curves are those reported in Table 1. As would be expected, whatever the extent of dehydration, the value of $t_{ext\%}$ decreases as the test temperature increases. This is not unexpected since dehydration is an activated process. In fact, by increasing the temperature, the phenomena involved accelerate; therefore, the time necessary to reach a certain level of dehydration decreases.

**Figure 9:**
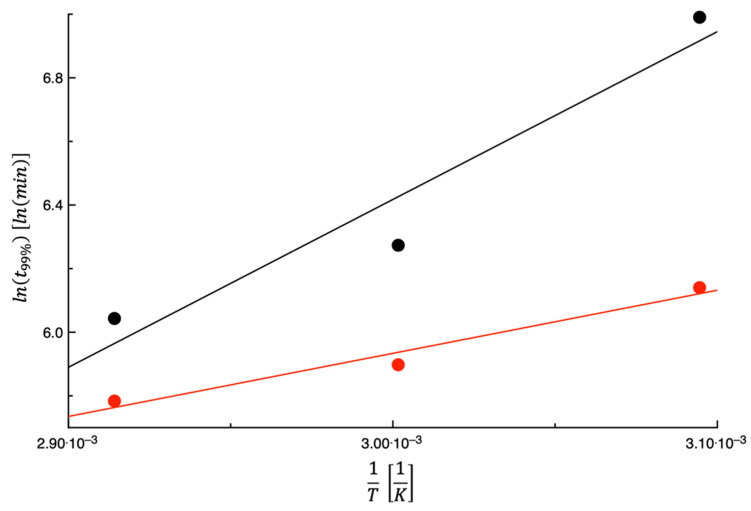
The expression $ln\left(t_{99\%}\right)$ was plotted as a function of $\frac{1}{T}$ for prickly pear peels (black) and tomato peels (red). The straight lines are obtained by fitting Equation (7) to the data.

According to Equation (5), the complete dehydration of the investigated PPPs (i.e., ext%=100%) occurs at infinite time; therefore, in this study, an ext%=99% has been used to compare the effect of drying temperature on $t_{ext\%}$. Consequently, the same procedure used to predict the curves shown in Figure 8 was used to calculate $t_{99\%}$ (i.e., the time needed to reach ext%=99%). Equation (7) was used to describe the dependence of $t_{99\%}$ on the testing temperature. In Figure 9, $ln\left(t_{99\%}\right)$ was plotted as a function of the inverse of the drying temperature. The curve shown in the same figure was obtained by fitting Equation (7) to the data. As performed before, in Table 2, the values of both Equation (7) parameters and $\bar{E}\%$ are listed. As expected, the Arrhenius-type equation satisfactorily fits the data; in fact, a value of $\bar{E}\%$ equal to 1.60 was obtained. As shown in the figure, $t_{99\%}$ steadly decreases as the temperature increases. As reported beforehand, this is not surprising, as the dehydration is an activated process.

The data of Lordi et al. [27] were used for comparative purposes, and they are also shown in Figure 9. As can be seen in the figure, at each temperature, the PPP takes more and more time to reach a degree of dehydration equal to 99%. This is not surprising considering that PPPs have a higher initial water content. Moreover, PPPs and TPSs have similar $K_{1}$, whereas the 2nd-stage dehydration rate of TPSs is higher than that of PPPs. Both factors mean that the time required for the dehydration of PPPs is greater than that required for TPSs.

### 3.3. Energy Consumption Calculation

**Figure 10:**
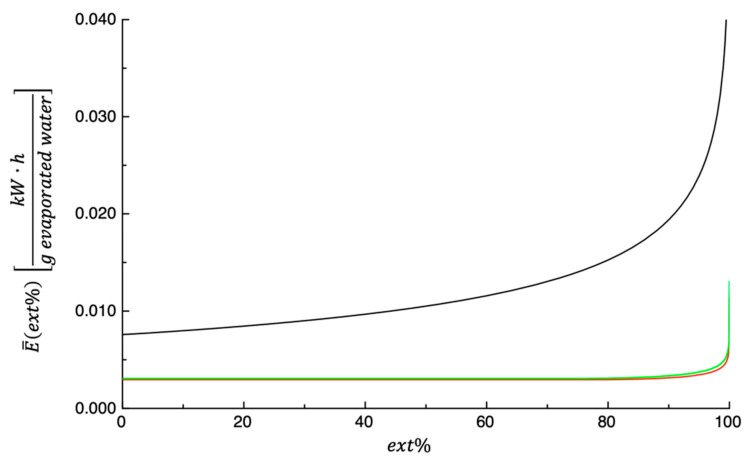
Prediction of $\bar{E}$ plotted as function of ext% at three different dehydration temperatures, 50 °C (black), 60 °C (red), and 70 °C (green).

The measured power supplied to the dehydration cabinet at the three tested temperatures was $0.00630\pm 2.76\cdot 10^{-6}\frac{kW\cdot h}{min\cdot 100\;g\;dry\;matter}$ at 50 °C, $0.00370\pm 8.96\cdot 10^{-6}\frac{kW\cdot h}{min\cdot 100\;g\;dry\;matter}$ at 60 °C, and $0.00525\pm 4.07\cdot 10^{-5}\frac{kW\cdot h}{min\cdot 100\;g\;dry\;matter}$ at 70 °C. Equations (18) and (19) were used to predict $\bar{E}$ vs. ext% (see Figure 10), and the values of parameters appearing in the above-mentioned equations were obtained by fitting the PPP dehydration kinetics (see Table 1), whereas the Θ values are those reported previously. As expected, $\bar{E}\left(ext\%\right)$ increases constantly with ext%, and the curves shown in the figure also indicate that the values of $\bar{E}\left(ext\%\right)$ obtained at 50 °C are always greater than those calculated at the other two temperatures, while the difference between the values obtained at 60 °C and 70 °C can be considered negligible.

**Figure 11:**
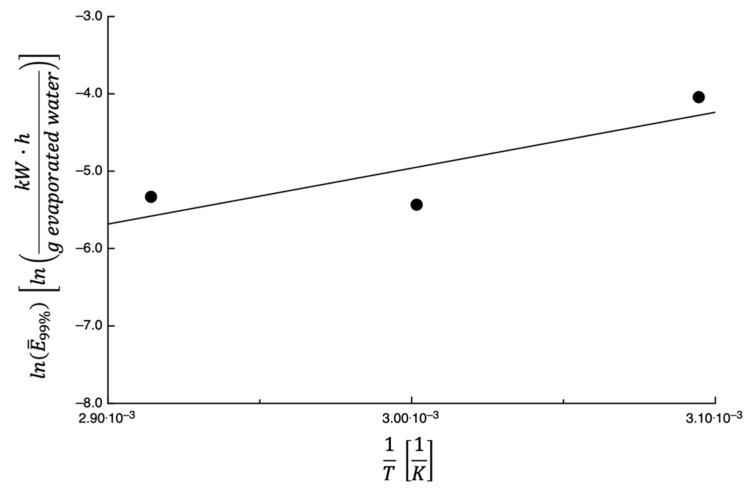
The expression $ln\left({\bar{E}}_{99\%}\right)$ is plotted as a function of $\frac{1}{T}$ for prickly pear peels. The straight line is obtained by fitting Equation (7) to the data.

As reported previously, a complete dehydration occurs at infinite time; therefore, ext%=99% was used to compare the effect of temperature on $\bar{E}\left(ext\%\right)$. The same procedure used to predict the curves shown in Figure 10 was used to calculate ${\bar{E}}_{99\%}$ (i.e., $\bar{E}\left(99\%\right)$). Equation (7) was used to describe the dependence of ${\bar{E}}_{99\%}$ on the dehydration temperature. In Figure 11, $ln\left({\bar{E}}_{99\%}\right)$ is plotted against the inverse of the temperature; the straight line was obtained by fitting Equation (7) to the data, and the parameter’s values obtained from fitting them together with the E% values are listed in Table 2. In this case, a slightly high value of E% has been obtained, probably due to the reduced number of available data and due to their dispersion.

## 4. Conclusions

This study was aimed at determining the optimal dehydration temperature of prickly pear peels (PPPs). The dehydration kinetics at three different temperatures along with the energy consumption of the dehydration process were measured. A mathematical model was used to fit the dehydration kinetics, and the dependence of the model’s parameters on the drying temperature was used to assess how the temperature affects the dehydration process. Data obtained were compared with data reported in the literature on the dehydration of tomato peels and seeds (TPSs). As expected, the dehydration rates of the 1st and 2nd stages (i.e., $K_{1}$ and $\frac{K_{1}}{K_{2}}$) were both an increasing function of the temperature; this is not surprising considering that dehydration is an activated process. The comparison between PPP and TPS data showed that $K_{1}$ of PPPs is like that of TPSs. This is not unexpected considering that the dehydration rate of the 1st stage is related to the evaporation of water from the surface and that for both by-products, we used the same dehydrating cabinet with the same set up. Considering the 2nd-stage dehydration rate, TPSs were found to have higher $\frac{K_{1}}{K_{2}}$ than PPPs. This is mainly because the latter has a thicker skin than that of tomatoes. $M_{H_{2}O}^{\infty }$ of the analyzed by-product was found to slightly increase with temperature, thus indicating that the water solubilization process is exothermic. On the contrary, data reported in the literature indicate that the water solubilization process in TPSs is endothermic. This is mostly because the two by-products have a different chemical composition, with PPPs being more hydrophilic, whereas TPSs have a predominant hydrophobic moiety. In terms of process productivity, it was found that $t_{ext\%}$ increased as the drying temperature increased. This is expected, as the dehydration process speeds up as the temperature increases. The term $t_{99\%}$ was used to compare data obtained in this work with what reported in the literature on TPSs. As expected, $t_{99\%}$ found for the TPSs was always lower than that of PPPs, considering that TPSs have a $\frac{K_{1}}{K_{2}}$ higher than PPPs, whereas the values of $K_{1}$ of the two by-products are similar.

The value of ${\bar{E}}_{99\%}$ was calculated to measure the energy consumption of the dehydration process. Data show that when increasing the temperature, a reduction of ${\bar{E}}_{99\%}$ can be obtained. If one considers that at a higher temperature, lower values of ${\bar{E}}_{99\%}$ and $t_{99\%}$ were obtained, it appears that among the dehydration temperatures investigated in this work, 70 °C is the optimal one.

## Figures and Tables

**Table 1:** Values of model’s parameters obtained by fitting the experimental data.

Parameters	50 °C	60 °C	70 °C
$t_{c}\;\left[min\right]$	258	282	202
$K_{1}\;\left[\frac{g\;desorbed\;water}{100\;g\;dry\;matter}\cdot \frac{1}{min}\right]$	0.830	1.25	1.71
$K_{2}\;\left[\frac{g\;desorbed\;water}{100\;g\;dry\;matter}\right]$	180	100	116

**Table 2:** Values of the parameters of Equation (7) obtained by fitting the experimental data.

Parameter	$ln\left(Y_{0}\right)$	$\frac{Y_{1}}{R}\left[K\right]$	$\bar{E}\%$
$t_{c}$	1.54	−1320	1.73
$K_{1}$	12.2	4009	2.44
$\frac{K_{1}}{K_{2}}$	14.8	6479	3.89
$M_{H_{2}O}^{\infty }$	8.74	888	0.434
$t_{99\%}$	−9.41	−5277	1.60
${\bar{E}}_{99\%}$	−26.6	−7228	6.47

## Data Availability

The original contributions presented in the study are included in the article, further inquiries can be directed to the corresponding author.
